# Scope of Policy Issues in eHealth: Results From a Structured Literature Review

**DOI:** 10.2196/jmir.1633

**Published:** 2012-02-17

**Authors:** Shariq Khoja, Hammad Durrani, Parvez Nayani, Ammad Fahim

**Affiliations:** ^1^AKDN eHealth Resource CentreThe Aga Khan UniversityNairobiKenya; ^2^Department of Community Health SciencesUniversity of CalgaryCalgary, ABCanada; ^3^VP Health & Operational Services DepartmentThe Aga Khan UniversityKabulAfghanistan; ^4^Programs in AfghanistanThe Aga Khan UniversityKabulAfghanistan; ^5^Department of Community Health SciencesThe Aga Khan UniversityKarachiPakistan

**Keywords:** eHealth, eHealth policies, telehealth, telemedicine, health informatics, electronic health records, health telematics, guidelines, standards

## Abstract

**Background:**

eHealth is widely used as a tool for improving health care delivery and information. However, distinct policies and strategies are required for its proper implementation and integration at national and international levels.

**Objective:**

To determine the scope of policy issues faced by individuals, institutions, or governments in implementing eHealth programs.

**Methods:**

We conducted a structured review of both peer-reviewed and gray literature from 1998–2008. A Medline search for peer-reviewed articles found 40 papers focusing on different aspects of eHealth policy. In addition, a Google search found 20 national- and international-level policy papers and documents. We reviewed these articles to extract policy issues and solutions described at different levels of care.

**Results:**

The literature search found 99 policy issues related to eHealth. We grouped these issues under the following themes: (1) networked care, (2) interjurisdictional practice, (3) diffusion of eHealth/digital divide, (4) eHealth integration with existing systems, (5) response to new initiatives, (6) goal-setting for eHealth policy, (7) evaluation and research, (8) investment, and (9) ethics in eHealth.

**Conclusions:**

We provide a list of policy issues that should be understood and addressed by policy makers at global, jurisdictional, and institutional levels, to facilitate smooth and reliable planning of eHealth programs.

## Introduction

eHealth policy can be defined as “a set of statements, directives, regulations, laws, and judicial interpretations that direct and manage the life cycle of eHealth” [1]. Recognition is growing in both developed and developing countries that eHealth is an important tool to reduce discrimination based on lack of access to information and to provide timely responses to matters affecting both personal and community health [2,3]. However, the use of eHealth within or between institutions involves several factors that require proper planning, supported by well-defined policies, rules, standards, or guidelines at the institutional, jurisdictional, and global levels. The absence of these policies may cause problems during the cycle of eHealth planning that may lead to failures in achieving the intended goals. As a result, there could be inadvertent widening of gaps in health status and knowledge levels between different sectors of the population, and increasing rather than decreasing health inequity, also termed the digital divide [4,5]. Experience from the developed world shows that the most common barriers to successful eHealth planning include lack of information on the role of eHealth in the provision of health care, lack of operational and support policies, lack of demonstrated cost effectiveness, and lack of clinical proponents [6].

To complement the need for eHealth policies and strategies within countries, pressure is also developing at the global level for eHealth policies. The World Health Assembly (WHA) resolution of 2005, WHA 58.28, calls on member states to draw up long-term strategic plans for the development and implementation of eHealth. Thus, it is important for the planners of eHealth at different levels to develop policies that could facilitate the adoption of eHealth and prove its success through improvement in services and change in the health status of the population. It is important for global forces, governments, and institutional leadership to understand the range of policy issues that must be addressed at different levels and stages of an eHealth program to facilitate its planning and implementation.

The objective of this study was to conduct a detailed review of the literature to determine the scope of policy issues faced by individuals, institutions, or governments in implementing eHealth programs. The study does not recommend any policies or suggest the importance of any of the policy issues over the others.

## Methods

We conducted a structured review of both peer-reviewed and gray literature. The search was conducted using the keywords *eHealth*, *telehealth*, *telemedicine*, *health informatics*, *electronic health records*, *health telematics*, *guidelines*, *policies*, *rules*, and *plans*. We collected the relevant information through the following process.

### Review of Peer-Reviewed Literature

We searched PubMed using the above-mentioned key words. We chose only English-language articles published in peer-reviewed journals during the 10-years period 1998–2008. The search yielded 950 articles. After removing duplicates and articles beyond the scope of this study, we selected 150 articles for the review. We developed our own lists of policy issues, which we used for the abstracts and full-text review. The review was conducted in two stages: (1) two of the researchers reviewed 150 abstracts to select the articles that were relevant and merited a review of the full paper according to the checklist, and (2) the same two reviewers then reviewed 40 full papers that focused on different aspects of eHealth policy, or highlighted the policy issues in eHealth implementation.

### Review of the Gray Literature:

We found 20 national- and international-level policy papers and documents through a Google search using the same key words described above. These articles were reviewed by two researchers to extract the policy issues and solutions described at different levels of care.

The list of issues was revised after the review. These issues were grouped into categories and themes for better understanding.

## Results

We extracted 99 policy issues related to eHealth from the literature. These issues were grouped under 9 themes on the basis of similarities in their application. We identified the following themes for eHealth policies: (1) networked care, (2) interjurisdictional practice, (3) diffusion of eHealth/digital divide, (4) eHealth integration with existing systems, (5) response to new initiatives, (6) goal-setting for eHealth policy, (7) evaluation and research, (8) investment, and (9) ethics in eHealth.

eHealth policy issues were also divided on the basis of the levels where policies should be developed to deal with a particular issue. The levels identified for the policy development were global, jurisdictional (national or provincial/subnational), and individual institutions. We used the following operational definitions for these levels: (a) global: this level deals with the policies of global complementarity, such as standardization and interjurisdictional care, (b) jurisdictional (national and provincial/subnational): this level deals with the policies required to facilitate care within a health jurisdiction—that is, national or provincial/subnational governments, and (c) individual Institutions: this level deals with the policies required to facilitate eHealth at the local level—that is, individual institution or practice.

Below we describe the distribution of eHealth policy issues according to the themes and the levels of policy development.

### Networked Care

The networked care theme [7-11] includes policy categories and issues that can enhance the ability of providers, departments, organizations, and jurisdictions to work in a coordinated environment to improve care of the population. Networked care covers the issues of interoperability [12,13], standardization [13], and intellectual property rights on material produced as a result of networked services [14], which need to be dealt with at the global level [12-14]. This theme also covers issues related to the use of acceptable, user-friendly, affordable, and reliable technology [14], to commitment to initial and ongoing funding [14,15], and to establishing local guidelines about sharing of information, standardization, communication, and control of malpractice [14-17], which can be dealt with at the jurisdictional level. The theme of networked care also includes issues related to change management, such as distribution of user workloads, improvement in readiness at the individual and institutional levels, and selection of simple and user-friendly technologies; financial matters, such as insurance requirements and reimbursement; guidelines related to sharing of information, knowledge, and services; cultural issues around communication and networking; and risk management [8,12-24]. Table 1 presents the matrix for the distribution of eHealth issues against the different levels under the theme of networked care.

**Table 1:** Networked care.

Level	Policy category	Issues	
a)	Global eHealth policies	i.	Functional and semantic interoperability
		ii.	Standardization of EHR^a^
		iii.	Intellectual property rights
b)	Jurisdictional (national and provincial/subnational) policies	i.	Regulation of appropriate technologies
		ii.	Commitment of funds
		iii.	Standardization of EHR
		iv.	Sharing of services
		v.	Proper connectivity
		vi.	Control of malpractice
		vii.	Cultural issues in communication
c)	Institutional/individual policies	i.	Proper distribution of human resources
		ii.	Readiness building and effective change management
		iii.	Deployment of appropriate technologies
		iv.	Meeting the needs of insurance companies
		v.	Reimbursement and remuneration
		vi.	Sharing of patient information
		vii.	Sharing of knowledge
		viii.	Sharing of services
		ix.	Standardization measures for EHR
		x.	Ensuring integrity and quality of data and information
		xi.	Proper connectivity
		xii.	Risk management
		xiii.	Cultural issues in communication

^a^ Electronic health record.

### Interjurisdictional Practice

The interjurisdictional practice theme [8] includes policy categories and issues that deal with the transfer of information and provision of care between different jurisdictions. Interjurisdictional practice includes issues related to management of health information in shared environments [25,26], policies for privacy, confidentiality, and intellectual property rights [1,8,13,14,27], and guidelines for sharing knowledge and services [8,16], which can be dealt with globally. Interjurisdictional practice also deals with policies at the jurisdictional and individual levels, such as liability of care [8,12,14], proper licensing of health care providers [8,12,28], accreditation of individuals and institutions, and the defining of processes for coordinated services [1,8]. Table 2 shows the policy issues that encompasses the theme of interjurisdictional practice.

**Table 2:** Interjurisdictional practice.

Level	Policy category	Issues	
a)	Global eHealth policies	i.	Policies on managing health information on the Internet
		ii.	Intellectual property rights
		iii.	Complementarity of policies and health care regulations in different regions
		iv.	Sharing of knowledge
b)	Jurisdictional (national and provincial/subnational) policies	i.	Accountability/liability of care
		ii.	Licensing
		iii.	Accreditation of services
		iv.	Local, national, and international policies
c)	Institutional/individual policies	i.	Accountability/liability of care

### Diffusion of eHealth and Addressing the Digital Divide

The diffusion of eHealth [29,30] and digital divide [8,31,32] theme includes policy categories and issues that enhance the use of eHealth among populations who most need improved health services. These include policies and guidelines to allow greater penetration of telecommunication companies, such as mobile companies, Internet service providers, integrated services digital network providers, and satellite vendors, to reach the poorest countries [8,13], reduce the cost of telecommunication [27], provide universal and unlimited access to the Internet [33], and allow for appropriate use of eHealth for commercial and humanitarian purposes [34]. Other policy issues with a jurisdictional and individual focus, such as encouraging development and use of open-source technologies, increasing access to technology, reducing cost, and building local capacity [35], are also included under this theme. Table 3 shows the list of policy issues covered under the theme of diffusion of eHealth.

**Table 3:** Diffusion of eHealth/digital divide.

Level	Policy category	Issues	
a)	Global eHealth policies	i.	Telecommunication policies allowing increased access
		ii.	Control of technology costs
		iii.	Provision of universal and unlimited access to the Internet
		iv.	Humanitarian vs commercial policies
		v.	Sharing of knowledge and services
b)	Jurisdictional (national and provincial/subnational) policies	i.	Increasing focus on open-source technologies
		ii.	Telecommunication policies allowing increased access
		iii.	Control of technology costs
		iv.	Capacity building
c)	Institutional/individual policies	i.	Capacity building

### Integration With Existing Systems

The theme of integration with existing systems [8,29] includes policy categories and issues that enable integration of eHealth projects and programs into regular services. The theme of integration includes jurisdictional policy issues such as setting targets for increasing interaction between different groups of providers and users, introducing decision-support systems to reduce the chance of errors [29], improving quality of care through eHealth and creating a learning environment, and changing business rules and models for integrating eHealth [33]. This theme also includes policies at the institutional level, such as defining the roles and responsibilities of different players [24], and creating guidelines on issues such as access to different gender and sociocultural groups, transfer and storage of information, patient consent, confidentiality, and privacy, which will help integrate eHealth with regular services [29,36].Table 4 lists the policy issues under the theme of integration with existing systems.

**Table 4:** eHealth integration with the existing systems.

Level	Policy category	Issues	
a)	Jurisdictional (national and provincial/subnational) policies	i.	Improvement of clinical effectiveness
		ii.	Improvement of quality of care
		iii.	Change in business rules in organizations
b)	Institutional/individual policies	i.	Redefinition of the roles and responsibilities of different players
		ii.	Wider ethical acceptability

### Response to New Initiatives

The response to new initiatives [29] theme includes policy categories and issues that can enhance the capability of institutions to implement eHealth successfully. This theme includes jurisdictional policy issues, such as guidelines for identifying and including stakeholders from different user and support groups in the planning of eHealth programs [24]. This theme also covers policy issues at the institutional level, such as defining the roles and responsibilities of different players such as local providers and specialists [24], defining the processes for change management [29], ensuring training and support to all users [29], defining the rules for procurement of equipment [13], distribution of bandwidth, and distribution and security of wireless networks [37], maintaining doctor–patient relationship [38], and evaluating new technologies in local environments before implementation to avoid difficulties and failure [29]. Table 5 lists the policy issues under the theme of response to new initiatives.

**Table 5:** Response to new initiatives.

Level	Policy category	Issues	
a)	Jurisdictional (national and provincial/subnational) policies	i.	Definition of stakeholders at different levels
b)	Institutional/individual policies	i.	Definition of the roles and responsibilities of different players, such as local providers and specialists
		ii.	Change management
		iii.	End-user support
		iv.	Regulation of information technology use
		v.	Maintenance of the doctor–patient relationships
		vi.	Wireless networks and security issues
		vii.	Evaluation of new technologies in local environments

### Policy Goal-Setting

The policy goal-setting [39] theme includes policy categories and issues that can guide the process of defining policies for eHealth. Key global considerations in this regard include recognition of eHealth as part of the broader development effort, in terms of assisting national health systems and recognizing eHealth as part of the global health agenda [40], and encouraging a global commitment for funding eHealth programs. This theme also includes jurisdictional considerations, such as developing policies to encourage growth of the telecommunications sector and to increase connectivity in remote areas [41]; increasing flexibility between governments and private institutions to align with changing information technology environments and policies [13]; encouraging innovation and development [38]; covering the costs of equipment and time needed for health care providers to bring eHealth services into broad acceptance [40]; and developing governance and management structures [41,42]. Institutional policy issues, such as ensuring universal standards of care, and allotting and distributing the workload for health care providers and technical and managerial staff [43], are also included under this theme. Table 6 lists the policy issues under the theme of goal-setting for eHealth policy.

**Table 6:** Goal-setting for eHealth policy.

Level	Policy Categories	Issues	
a)	Global eHealth policies	i.	Integration of eHealth into the overall development effort
		ii.	Funding of eHealth programs
b)	Jurisdictional (national and provincial/subnational) policies	i.	Provision of suitable telecommunications infrastructure to promote eHealth
		ii.	Alignment of policies with information technology innovations
		iii.	Innovative and forward-looking policies
		iv.	Coverage of the opportunity cost of health providers’ time
		v.	Timing of government action
		vi.	Development of leadership structures for eHealth programs
		vii.	Development of strategies for eHealth adoption
		viii.	Information governance
c)	Institutional/individual policies	i.	Standards of care
		ii.	Guidelines for human resources

### Evaluation and Research

The evaluation and research [39] theme includes policy categories and issues that can guide the process of evaluation and research to generate evidence for the adoption of eHealth. These policy issues include measurement of the time spent during teleconsultations and justification of the resources spent on setting up eHealth services [44], cost effectiveness [45], impact on health care management [45], demonstration of improvement in health outcomes [46], and enhancement of clinical effectiveness [38] and learning [3]. Other issues at the level of individual institutions include providing an environment for testing and simulating eHealth initiatives [37], encouraging interdisciplinary research [47], and disseminating results for policy making and the benefit of users [3]. Table 7 lists the policy issues under the theme of evaluation and research.

**Table 7:** Evaluation and research.

Level	Policy category	Issues	
a)	Jurisdictional (national and provincial/subnational) policies	i.	Justification of health providers’ time
		ii.	Cost effectiveness
		iii.	Impact of eHealth on health care management
		iv.	Demonstration of health outcomes
		v.	Evidence of clinical effectiveness
		vi.	Progress in learning
b)	Institutional/individual policies	i.	Provision of simulation environment
		ii.	Encouragement of coordinated research
		iii.	Dissemination for policy making and benefit of others

### Investment

The investment [9] theme includes policy issues that can suggest business models for eHealth adoption. This theme includes encouraging the use of eHealth by health care institutions to increase the number of clients and to grow their businesses [14] and encouraging partnerships between public and private institutions, or within the same sector. It also includes cross-jurisdictional advertisement and sale of drugs and services. Table 8 lists the policy issues under the theme of investment.

**Table 8:** Investment.

Level	Policy category	Issues	
a)	Jurisdictional (national and provincial/subnational) policies	i.	Use of eHealth for commercial purposes
		ii.	Public–private partnership
		iii.	Cross-border advertisement and sale of drugs

### Ethics and Legal Issues in eHealth

The theme of ethics and legal issues in eHealth [25] includes the ethical issues that must be considered during adoption of eHealth. These include global policy issues, such as managing health information on the Internet [25,26] and ensuring privacy of health information [27]. This theme also includes jurisdictional and institutional policy issues, such as patient consent [24,48], liability of care [45,48-50], medicolegal issues [45], patients’ rights to access their own health information [14], security of information during portability [48], maintenance of quality of care [14,17], and cultural issues in communication [14]. Table 9 lists the policy issues under the theme of ethics and legal issues in eHealth.

**Table 9:** Ethical and legal issues in eHealth.

Level	Policy category	Issues	
a)	Global eHealth policies	i.	Management of health information on the Internet
		ii.	Health information privacy
b)	Jurisdictional (national and provincial/subnational) policies	i.	Consent for care in eHealth
		ii.	Liability issues (medical malpractice liability)
		iii.	Medicolegal issues
		iv.	Patients’ right to access information
		v.	Security of information during portability
		vi.	Control of malpractice
		vii.	Cultural issues in communication

## Discussion

This policy paper provides a spectrum of eHealth issues that require policies for different levels of decision makers. It is important for the policy makers at the global, national, and institutional levels to understand the scope and importance of these issues; to analyze their current situation; and to take a proactive approach to developing policies that facilitate smooth and reliable planning of eHealth programs.

Based on our findings, we recommend a combination of policies at different levels when developing eHealth policies. Many strategies suggest that the development of supportive policies should be part of the eHealth strategies of countries and organizations. These recommendations should, however, come from the user groups and managers of eHealth programs in each country. It is therefore important to increase the awareness that health care providers, managers, and policy makers at all levels have of eHealth policy issues by providing them guidelines and support to develop these policies.

The main strength of this paper is that it draws on policy issues from both peer-reviewed and gray literature. Our review of policy papers provided a detailed analysis of different policy issues and their relationship with other such issues at different levels. Due to the limitation of time and resources, it was not possible to review the literature in languages other than English, or to conduct a detailed systematic review.

As a follow-up to this study, an environmental scan at all three levels (global, national, and institutional) should be conducted to identify and study the already-existing policies on the issues identified in this paper. There is a need to study the successes and failures of these policies, which will support the development of guidelines for policy makers at the global, regional, national, and local levels. This would lead to policy formulation that not only benefits their own eHealth programs but also generates knowledge to support programs in other areas.

Finally, we have also developed a 2-way matrix that will show which policies are relevant at different stages of eHealth planning, implementation, integration, and sustainability. The findings of this exercise merit a separate publication, which should help eHealth planners to assess the need for relevant policies for different eHealth initiatives and to develop the policy issues most relevant to that stage.

## Abbreviations

WHA: World Health Assembly
